# Determinants of Antimicrobial Resistance in *Escherichia coli* Strains Isolated from Faeces and Urine of Women with Recurrent Urinary Tract Infections

**DOI:** 10.1371/journal.pone.0049909

**Published:** 2012-11-16

**Authors:** Casper D. J. den Heijer, Mariëlle A. J. Beerepoot, Jan M. Prins, Suzanne E. Geerlings, Ellen E. Stobberingh

**Affiliations:** 1 Department of Medical Microbiology, Maastricht University Medical Centre/Care and Public Health Research Institute (CAPHRI), Maastricht, The Netherlands; 2 Division of Infectious Diseases, Department of Internal Medicine, Centre for Infection and Immunity Amsterdam (CINIMA), Academic Medical Centre, Amsterdam, The Netherlands; University of Hyderabad, India

## Abstract

For women with recurrent urinary tract infections (rUTI), the contribution of antibiotic use versus patient-related factors in determining the presence of antimicrobial resistance in faecal and urinary *Escherichia coli*, obtained from the same patient population, has not been assessed yet. Within the context of the ‘Non-antibiotic prophylaxis for recurrent urinary tract infections’ (NAPRUTI) study, the present study assessed determinants of antimicrobial resistance in *E. coli* isolated from urinary and faecal samples of women with rUTIs collected at baseline. Potential determinants of resistance were retrieved from self-administered questionnaires. From 434 asymptomatic women, 433 urinary and 424 faecal samples were obtained. *E. coli* was isolated from 146 (34%) urinary samples and from 336 (79%) faecal samples, and subsequently tested for antimicrobial susceptibility. Multivariable analysis showed trimethoprim/sulfamethoxazole (SXT) use three months prior to inclusion to be associated with urine *E. coli* resistance to amoxicillin (OR 3.6, 95% confidence interval: 1.3–9.9), amoxicillin-clavulanic acid (OR 4.4, 1.5–13.3), trimethoprim (OR 3.9, 1.4–10.5) and SXT (OR 3.2, 1.2–8.5), and with faecal *E. coli* resistance to trimethoprim (OR 2.0, 1.0–3.7). The number of UTIs in the preceding year was correlated with urine *E. coli* resistance to amoxicillin-clavulanic acid (OR 1.11, 1.01–1.22), trimethoprim (OR 1.13, 1.03–1.23) and SXT (OR 1.10, 1.01–1.19). Age was predictive for faecal *E. coli* resistance to amoxicillin (OR 1.02, 1.00–1.03), norfloxacin and ciprofloxacin (both OR 1.03, 1.01–1.06). In conclusion, in women with rUTI different determinants were found for urinary and faecal *E. coli* resistance. Previous antibiotic use and UTI history were associated with urine *E. coli* resistance and age was a predictor of faecal *E. coli* resistance. These associations could best be explained by cumulative antibiotic use.

## Introduction

The association between antibiotic use and antimicrobial resistance has been convincingly demonstrated [1]–[3]. On the individual patient level, this is a clinical problem in patients with urinary tract infections (UTI), in particular in women with recurrent UTI (rUTI). The (recurrent) empirical antimicrobial treatment in these women exerts significant resistance pressure on the uropathogens [4]. This pressure also affects the faecal flora, which serves as a resistance reservoir for potential uropathogens [5], [6].

Besides antibiotic use, patient-related factors could be predictive for the presence of resistant uropathogens. For women with rUTI, a higher prevalence of resistance has been observed in women who had complicating host factors compared with women without these factors [3]. Knowledge on the predictors of antimicrobial resistance can be helpful for clinicians to determine the optimal empirical therapy for women with rUTI.

The contribution of antibiotic use versus patient-related factors as determinants of antimicrobial resistance in faecal and urinary *E. coli*, obtained from the same patient population, has not been assessed yet. We recently reported the results of the ‘Non-antibiotic prophylaxis for recurrent urinary tract infections’ (NAPRUTI) study: two studies on antibiotic versus non-antibiotic prophylaxis in pre- and postmenopausal women with rUTI [2], [3]. We here investigated the determinants of resistance in the faecal and urinary *E. coli* isolates obtained at baseline in these women.

## Materials and Methods

### Patients

This study was conducted in the context of the ‘Non-antibiotic prophylaxis for recurrent urinary tract infections’ (NAPRUTI) study [2], [3]. The study consisted of two randomized controlled multicentre trials comparing cranberries or lactobacilli with trimethoprim/sulfamethoxazole (SXT) prophylaxis in pre- and postmenopausal women with recurrent UTIs respectively. Eligible for inclusion were non-hospitalized (both primary care and out-patient clinic) women over 18 years who had experienced three or more symptomatic UTIs in the year preceding enrolment. Patients were excluded when symptoms of UTI were noted at baseline and when any antibiotic had been taken in the previous two weeks. For the present analysis, women were eligible with a urine and/or faecal sample available at NAPRUTI baseline.

### Ethics Statement

The study protocol was approved by the Medical Ethics Committees of all participating Centres, ie. Academic Medical Centre (Amsterdam), Maastricht University Medical Centre, Maasland hospital (Sittard), Onze Lieve Vrouwe Gasthuis (Amsterdam), Sint Lucas Andreas hospital (Amsterdam), Slotervaart hospital (Amsterdam), University Medical Centre Utrecht, Medical Centre Alkmaar, Haga hospital (The Hague) and Scheper hospital (Emmen). All participants provided written informed consent prior to inclusion.

### Determinants

A baseline questionnaire was completed by all subjects (n = 434), including information on the following potential determinants of antimicrobial resistance: the number of UTI episodes in the previous 12 months, antibiotic use in the previous three months (yes/no and if yes subdivided into 4 groups: trimethoprim or SXT; amoxicillin or amoxicillin-clavulanic acid; nitrofurantoin; quinolones), age (in years), and the presence or absence of complicating host factors. These factors were defined as having a history of functional or structural abnormalities of the urinary tract (yes/no); diabetes mellitus (yes/no); the use of a urinary catheter (yes/no); or of immunosuppressive medication (yes/no). Women who had at least one complicating host factor were classified as having complicating host factors; the remaining women as having no complicating host factors.

### Urinary and Faecal Isolates

Midstream urinary and faecal samples were collected at study baseline. Dipslides (Uriline, 56508, Biomérieux, Plainview, NY, USA) were prepared from collected urinary samples and sent, together with the faecal samples, to the microbiological laboratory of Maastricht University Medical Centre for identification of the microorganisms and antimicrobial susceptibility testing. Bacteriological analysis on faecal samples was done as previously described [7]. Only the predominant *E. coli* strain of each sample was included in the final analysis.

### Antimicrobial Susceptibility Testing

Antimicrobial susceptibility of the *E. coli* isolates was determined in accordance with the European Committee on Antimicrobial Susceptibility Testing (EUCAST) guidelines using the microdilution method [8]. The testing included the following agents: amoxicillin, amoxicillin-clavulanic acid, trimethoprim, trimethoprim/sulfamethoxazole (SXT), norfloxacin, ciprofloxacin and nitrofurantoin.

### Statistical Analysis

For each antimicrobial agent for which the *E. coli* susceptibility was tested, determinants of antimicrobial resistance were analysed. Crude and adjusted odds ratios (ORs) were calculated for the association between antimicrobial resistance and each determinant. Age was considered as a continuous variable. For the calculation of ORs of the association between antimicrobial resistance and (specific) antimicrobial use, women who had not taken any antimicrobial agent in the previous three months were used as control group. For the calculation of the adjusted ORs, all determinants were included in a logistic regression model. SPSS 16.0 was used for statistical analyses and a P-value of<0.05 was considered statistically significant.

## Results

### Patients

In total, 433 urinary and 424 faecal samples were obtained from 434 women. 279/433 (64%) urinary samples yielded a uropathogen, of which 146 (52%) were *E. coli*. From 336 faecal samples *E. coli* was isolated (79%). The baseline characteristics of the women from whom an *E. coli* strain was isolated are given in Table 1.

**Table 1:** Baseline characteristics of women from whom an *Escherichia coli* strain was isolated, stratified for the origin of the sample.

	Sample in which *E. coli* strain was isolated		Total study population
	Urine (n = 146)	Faeces (n = 336)	(n = 434)
**Age, mean (SD)**	52.2 (17.1)	49.9 (17.4)	50.8 (17.4)
**Number of UTIs in year preceding enrolment,** **mean (SD)**	7.0 (4.5)	6.8 (3.8)	6.8 (3.9)
**Presence of complicating host factors**	49 (33.6)	91 (27.2)	126 (29.0)
**Postmenopausal**	82 (56.2)	172 (51.3)	233 (53.7)
**Antibiotics used in previous three months:**			
**- Any**	115 (78.8)	255 (76.1)	339 (78.1)
**- Amoxicillin or amoxicillin-clavulanic acid**	18 (12.3)	32 (9.6)	38 (8.8)
**- Trimethoprim or SXT**	22 (15.1)	56 (16.7)	70 (16.1)
**- Nitrofurantoin**	55 (37.7)	117 (34.9)	151 (34.8)
**- Quinolones**	22 (15.1)	53 (15.8)	78 (18.0)

**NOTE.** SD = standard deviation, SXT = trimethoprim/sulfamethoxazole. Numbers are n (%), unless otherwise stated.

### Antimicrobial Susceptibility

The antimicrobial resistances of the urinary respectively faecal *E. coli* strains were: amoxicillin: 34%/29%, amoxicillin-clavulanic acid: 17%/10%, trimethoprim: 30%/24%, SXT: 28%/22%, norfloxacin: 14%/9%, ciprofloxacin: 14%/9%, and nitrofurantoin: 0%/1%.

Urinary *E. coli* were significantly more often resistant to amoxicillin-clavulanic acid than faecal *E. coli* (OR 1.90, 95% confidence interval (CI): 1.08–3.32). All other resistance percentages were not significantly different between urinary and faecal isolates. Because of the low prevalence of resistance to nitrofurantoin, this agent was excluded from further analysis.

### Determinants of Antimicrobial Resistance

The significant associations between *E. coli* resistance and included potential determinants are given in Table 2. The use of trimethoprim or SXT in the previous three months was associated with increased urinary *E. coli* resistance to amoxicillin, amoxicillin-clavulanic acid, trimethoprim and SXT. Urinary *E. coli* resistance to the latter three agents was also related to the number of UTIs in the year preceding enrolment, and the presence of complicating host factors was predictive for amoxicillin-clavulanic acid resistance.

**Table 2:** Significant determinants of urinary and faecal *Escherichia coli* antimicrobial resistance.

Origin	*E. coli* resistance to	Determinant	Crude OR (95% CI)^a^	Adjusted^b^ OR (95% CI)^a^
**Urine**	Amoxicillin	SXT use	4.2 (1.3–13.4)	3.6 (1.3–9.9)
	Amoxicillin-clavulanic acid	SXT use	6.5 (1.5–27.9)	4.4 (1.5–13.3)
		UTI history	1.13 (1.04–1.23)	1.11 (1.01–1.22)
		Complicating host factors	4.7 (1.9–11.8)	4.0 (1.4–11.7)
	Trimethoprim	SXT use	4.1 (1.3–13.5)	3.9 (1.4–10.5)
		UTI history	1.12 (1.04–1.21)	1.13 (1.03–1.23)
	SXT	SXT use	3.4 (1.0–11.2)	3.2 (1.2–8.5)
		UTI history	1.10 (1.00–1.03)	1.10 (1.01–1.19)
**Faeces**	Amoxicillin	Age	1.01 (1.00–1.03)	1.02 (1.00–1.03)
	Trimethoprim	SXT use	3.9 (1.6–9.2)	2.0 (1.0–3.7)
	Ciprofloxacin	Age	1.04 (1.01–1.06)	1.03 (1.01–1.06)
	Norfloxacin	Age	1.04 (1.01–1.06)	1.03 (1.01–1.06)

**NOTE.** OR = odds ratio, 95% CI = 95% confidence interval, SXT = trimethoprim/sulfamethoxazole.

a Women who had not taken any antimicrobial agent in the previous three months were used as reference group for the calculation of ORs of the association between antimicrobial resistance and (specific) antimicrobial use.

b Adjusted for age, UTI history, antibiotic use in the previous three months and presence or absence of complicating host factors.

In faecal *E. coli*, age was positively associated with resistance to amoxicillin and fluoroquinolones and the use of trimethoprim or SXT was predictive for trimethoprim resistance.

## Discussion

In women with rUTI, different determinants were found for antibiotic resistance in urinary and faecal *E. coli* isolates. For urinary *E. coli* resistance, the use of TMP or SXT and a history of UTI were the most important determinants, whereas patient’s age was the most determinative for faecal *E. coli* resistance.

To our knowledge, this is the first study that assessed the contribution of antibiotic use versus patient-related factors as determinants of antimicrobial resistance in *E. coli* isolated from both urinary and faecal samples, for women with rUTI.

In patients with acute febrile infections, Raum and colleagues have shown that SXT use influences the prevalence of *E. coli* resistance in faecal samples, even two weeks after cessation of therapy. For beta-lactam antibiotics and doxycycline, this effect was not observed [5]. Likewise, in the present study, in which antimicrobial use had to be stopped two weeks prior to inclusion, *E. coli* resistance was only associated with the use of trimethoprim or SXT. This suggests that the relationship between antimicrobial use and antimicrobial resistance is stronger for SXT than for other agents.

In a meta-analysis on the relationship between antibiotic use and resistance, Costelloe et al. found weak but detectable associations 12 months after exposure. They argued that this residual effect is likely to be an important driver for the high endemic levels of antimicrobial resistance in the community [9]. The isolated faecal *E. coli* is considered to be the predominant strain of the patient’s commensal flora [10], and is exposed to (orally taken) antimicrobial agents during a patient’s lifetime, resulting in gradually increasing resistance with increasing age, as found in the present study.

The specific pathogenesis of rUTI, with increasing evidence of the existence of biofilm-like communities in the bladder from which bacteria are released to cause rUTI, makes resistance of urinary *E. coli* from women with rUTI possibly less dependent on age [11]. In this respect, the lifespan of the biofilm could be of more importance. It has been suggested that the biofilm reduces the susceptibility of *E. coli in vivo* by ineffective antimicrobial diffusion and alterations in the metabolic state of biofilm-associated strains [11]. This might explain the overall trend of higher resistance in urinary compared with faecal *E. coli* found in this study, being however significant for amoxicillin-clavulanic acid only.

In accordance with Costelloe, we found an association between recent antibiotic exposure and resistance in urine [9]. The increased prevalence of resistance to amoxicillin and amoxicillin-clavulanic acid after SXT use could be explained by the fact that resistance genes for these antimicrobials are located on the same plasmid [12].

Another predictor of urinary antimicrobial resistance, the number of UTI episodes in the previous year, is probably associated with prior antibiotic use, as previously suggested [13].

Dutch UTI guidelines for general practitioners recommend amoxicillin-clavulanic acid treatment in patients with complicating host factors [14]. The detected association between amoxicillin-clavulanic acid resistance and the presence of complicating host factors might be attributable to the previous use of this antimicrobial agent in patients with these factors.

A limitation of the study was that antimicrobial use in the previous three months was retrieved from self-administered questionnaires as well as the number of UTIs in the year preceding enrolment. This can make these determinants prone to misclassification bias. However, women were blinded for the resistance status of their *E. coli* isolates, making differential misclassification less likely.

A number of stool cultures did not yield *E. coli* (21%). This indicates that in these samples fewer than 300 CFU of *E. coli* (minimum detection level) were present per gram faeces, as reported previously by a study that used the same bacteriological analysis [7].

In addition, all included women were asymptomatic, which could limit the translation of our results to women with symptomatic UTI. However, we have recently shown that asymptomatic *E. coli* strains are predictive for strains that cause a symptomatic *E. coli* UTI in women with rUTI [15].

The associations between patient’s age and faecal *E. coli* resistance seem marginal, although it needs to be taken into account that age was considered a continuous variable in all analysis. So, with each increase of one year in age, a women with rUTI has a 1.03 times higher chance of having a ciprofloxacin-resistant faecal *E. coli*. This translates to a 6 times higher chance for a 80-year old women compared with a 20-year old, showing the clinical relevance of this observation.

No genotyping has been performed on the *E. coli* isolates in the present study. However, our aim was to provide clinicians the knowledge on predictors of antimicrobial resistance, which could be helpful when treating a women with rUTI empirically. At that moment, clinicians have no information on the genotypic background of the causative uropathogen at their disposal.

Concluding, in women with rUTI, differences were observed in determinants of urinary versus faecal *E. coli* resistance, which could be attributed to the specific pathogenesis of rUTI. The observed determinants of resistance can best be explained by cumulative antibiotic use.
